# Picture quiz

**Published:** 2023-05-22

**Authors:** Louis Oteng-Gyimah, Victor H Hu

**Affiliations:** Ophthalmologist: Sunyani SDA Hospital, Sunyani, Ghana.; Assistant Clinical Professor: International Centre for Eye Health, London School of Hygiene & Tropical Medicine and Consultant Ophthalmologist: Mid Cheshire NHS Hospitals, UK.

**This quiz is based on a real patient. Read the information, then use your knowledge and clinical skills to answer the questions. We suggest you use a separate sheet of paper, then compare your answers with those provided at the bottom of the page.**

**Figure 1:**
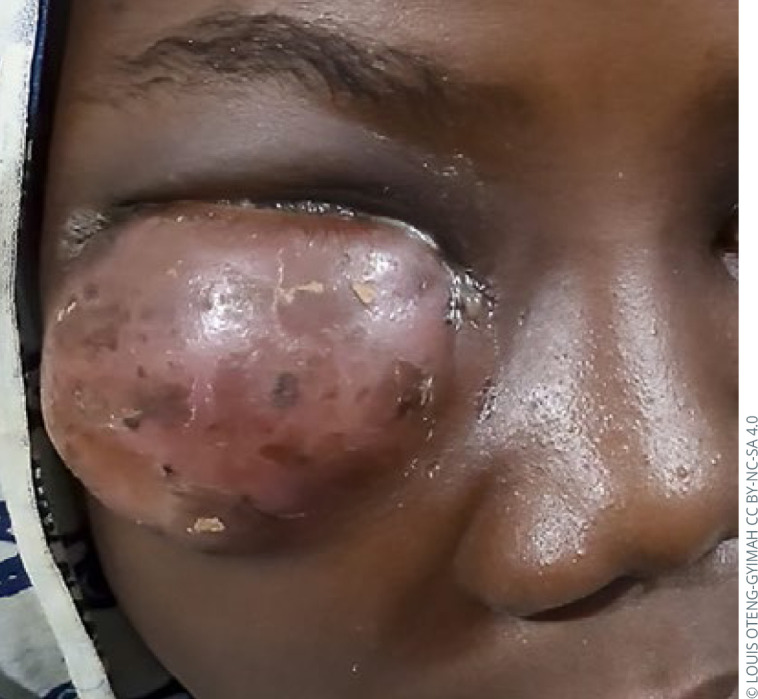
Swelling of the right eyelid in a 14-year-old. ghana

A 14-year-old girl presented with painful swelling of her right lower eyelid. The swelling started seven days earlier, after the eyelid was scratched by a tree branch. There was no other past ocular or medical history. The vision in the left eye was 6/6 unaided, but vision in the right eye could not be assessed because of the swelling.

**Learning outcome**

By the end of this quiz, you will be able to reflect on your clinical approach and strategy for managing cases, as well as other unusual ocular presentations.

**Question 1 What are your initial thoughts on seeing this presentation?**
**Question 2 What could you consider in your differential diagnosis?**
**Question 3 Are there any other examination findings or tests you would like to do?**
**Question 4 Based on your diagnosis, what immediate treatment would you start for this patient?**
  □ **a.** Monitor over the next few days to see how she progresses
  □ **b.** Antibiotic eye ointment
  □ **c.** Urgent incision and drainage
  □ **d.** Start with oral antibiotics and ask her to come back in three days.
**Question 5 When considering the differential diagnosis of any presentation it can be helpful to use a mnemonic (a memory aid). One example of this is the phrase ‘Vitamin C & D.’ Each letter in this phrase corresponds to the name of a type of disease process that should be considered when assessing an unusual presentation, such as the one in this quiz.** (Note: there can be more than one condition for some of the letters.)
  **Table:** 

**V**	Vascular disease (i.e., to do with blood vessels and blood supply)
**I**	Infectious or inflammatory
**T**	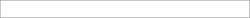
**A**	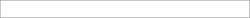
**M**	Metabolic
**I**	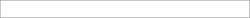
**N**	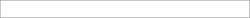
**C**	Congenital (present from birth)
&	
**D**	Degenerative

## ANSWERS

**Figure 2:**
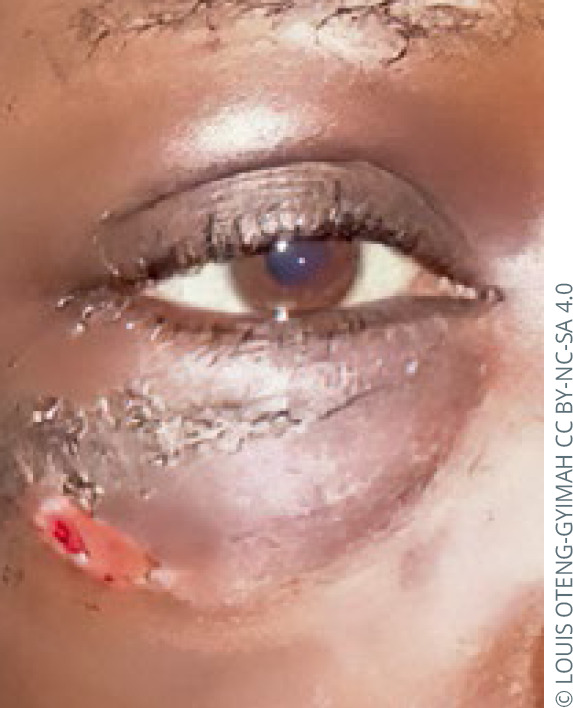
The same eye following treatment. GHANA

1. There is a very large swelling of the lower lid, which looks likely to be tense and very painful. The history is important: it has only been there for 7 days. It also occurred after some trauma, which makes the diagnosis most likely to be some sort of infection.
2. Lid swelling can be caused by tumours, but these would usually develop more slowly, over weeks to months (although Merkel cell tumours can develop more rapidly). Vascular disease, such as haemangiomas, can cause lid swelling; however, they would not be expected to develop this rapidly or look like this.
3. It would be helpful to look for swollen lymph glands (lymphadenopathy), e.g., in front of the ear and under the jaw, as this would be consistent with an infection. Other symptoms which would suggest an infection include a raised temperature and a raised white cell count. Some form of imaging scan (e.g., CT or MRI) might be useful to gain further information about the swelling, such as whether it extends back behind the eyeball. However, this would depend on the availability of these tests; they are probably not necessary at this stage as this is most likely an infection and treatment should be initiated without delay. Note that people living with HIV may be more likely to develop severe bacterial infections; a test may be indicated in order that treatment can be initiated to prevent future infections.
4. c. This child has a large abscess of the right lower lid. An abscess of this size requires incision and drainage. This would be done using local anaesthetic over the incision site (given very slowly, as the area will be very tender). A large amount of pus will need to be drained. A course of systemic antibiotics should also be used to help treat the infection.
5. The letters in Vitamin C & D correspond to the following:
  V: vascular disease (i.e., to do with blood vessels and blood supply)
  I: infectious or inflammatory
  T: traumatic or toxic
  A: autoimmune or allergy
  M: metabolic
  I: iatrogenic (caused by treatment), idiopathic (cause unknown), or inherited (genetic in origin)
  N: neoplastic (i.e., tumours or cancer)
  C: congenital (present from birth)
  &
  D: degenerative
  Remember that there are two T's and five I's altogether.

